# Dexmedetomidine activates the PKA/CREB pathway and inhibits proinflammatory factor expression through β2 adrenergic receptors

**DOI:** 10.1002/iid3.1176

**Published:** 2024-02-27

**Authors:** Baocheng Zhang, Jie Shen

**Affiliations:** ^1^ Department of Emergency and Critical Care Center Jinshan Hospital Affiliated to Fudan University Shanghai China

**Keywords:** dexmedetomidine, IL‐10, inflammatory factor, β2 adrenergic receptor

## Abstract

**Introduction:**

Dexmedetomidine (DEX) is primarily utilized for sedation in the context of general anesthesia or intensive care. However, the exact regulatory mechanism by which DEX affects cytokines remains unclear. This study aims to investigate the underlying mechanism by which DEX inhibits proinflammatory factors through activation of the β2 adrenergic receptor (β2 AR).

**Methods:**

The inflammatory cell model of human mononuclear macrophage (THP‐1) cells induced by lipopolysaccharide (LPS) was established to study the effect of DEX on the expression of cell‐related inflammatory factors. ADRA2A gene knockout THP‐1 cells (THP‐1^KO^) were constructed by CRISPR technology, and the effect of DEX on the expression of inflammatory factors of THP‐1^KO^ cells was detected. The target sites of DEX on β2 AR were screened by molecular docking. Reversion experiments were performed using ADRB2‐siRNA. Western blot was used to detect the activation of β2 AR/PKA/CREB pathway and NF‐κB, and ELISA was used to detect the release level of inflammatory factors.

**Results:**

The results demonstrated a significant reduction in the expression levels of MCP‐1, interleukin‐06, and IL‐8 in both THP‐1 and THP‐1^KO^ cells when induced by LPS following treatment with DEX. Additionally, DEX treatment led to an increase in IL‐10 expression. Immunofluorescence analysis revealed an upregulation of β2 AR expression after DEX treatment. Western blot results indicated that DEX notably enhanced the activation of the β2 AR and PKA/CREB pathways, while concurrently suppressing the activation of NF‐κB. Notably, the use of ADRB2 siRNA reversed the effects of DEX treatment and promoted the release of inflammatory cytokines.

**Conclusion:**

DEX initiates the activation of the PKA/CREB pathway through the activation of β2 AR. Simultaneously, it exerts an inhibitory effect on the activation of NF‐κB, consequently reducing the transcription of proinflammatory factors while increasing the transcription of anti‐inflammatory factors.

## Graphical abstract

The inflammatory cell model of human mononuclear macrophage (THP‐1) cells induced by lipopolysaccharide (LPS) was established to study the effect of Dexmedetomidine (DEX) on the expression of cell‐related inflammatory factors. ADRA2A gene knockout THP‐1 cells (THP‐1KO) were constructed, and the effect of DEX on the expression of inflammatory factors of THP‐1KO cells was detected. The target sites of DEX on β2 AR were screened by molecular docking. Reversion experiments were performed using ADRB2siRNA. The results showed that DEX significantly decreased the expression of MCP‐1, interleukin‐06, and IL‐8 in THP‐1 and THP‐1KO cells induced by the LPS, and increased the level of IL‐10. Immunofluorescence results showed that β2 adrenergic receptor (β2 AR) expression increased after DEX treatment. DEX significantly increased the activation of β2 AR and PKA/CREB pathways, inhibited the activation of NF‐κB. ADRB2 siRNA reversed the effects of DEX and promoted the release of inflammatory cytokines

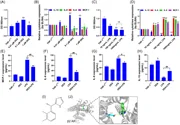

## 1 INTRODUCTION

Dexmedetomidine (DEX) is a rapidly acting and highly selective α2 adrenoceptor (α2 AR) agonist.^1^, ^2^ Its primary clinical applications include sedation during general anesthesia and in intensive care settings.^3^ Previous research has highlighted several beneficial effects of DEX in various medical contexts. For instance, in severe acute pancreatitis, DEX has been shown to reduce the infiltration of neutrophils and macrophages while mitigating oxidative stress.^4^ Moreover, DEX has demonstrated the ability to protect against pituitary damage induced by X‐ray radiation by preventing cell apoptosis.^5^ Additionally, DEX exhibits superior efficacy in managing mental symptoms such as anxiety and delirium following various surgical procedures, which may be attributed to its anti‐inflammatory properties.^6^ Numerous studies have established that DEX can modulate cytokine secretion. Notably, DEX significantly inhibits the release of inflammatory factors post‐surgery, including C‐reactive protein, interleukin‐1β, interleukin‐06 (IL‐6), and tumor necrosis factor‐alpha.^7^, ^8^, ^9^ By reducing the inflammatory response, DEX alleviates patient discomfort, improves prognosis, and shortens hospitalization durations.^10^ In a rat cecal ligation and puncture (CLP) model, DEX has been found effective in reducing the production of inflammatory mediators in both plasma and bronchoalveolar lavage fluid in septic rats induced by CLP.^11^ However, the precise mechanisms underlying DEX's regulation of cytokines require further elucidation.

To further study the regulatory effect of DEX on inflammatory factors, this study took the human mononuclear macrophage (THP‐1) inflammatory model as the research object to study the effects of DEX on IL‐6, IL‐8 and MCP‐1 of THP‐1 cells after lipopolysaccharide (LPS) induction, as well as the effects on the expression of IL‐10. The main target of DEX, ADRA2A, was knocked out by CRISPR technology, the expression of relevant receptors was detected by immunofluorescence and molecular docking to clarify the mechanism of DEX regulation of inflammatory factors.

## 2 MATERIALS AND METHODS

### 2.1 Cell culture

The human THP‐1 cell line was purchased from Zhejiang Ruyao Biotechnology Co., Ltd. The culture conditions were DMEM (PM150210) + 10% FBS (164210‐500) + 1% penicillin‐streptomycin double antibody (PB180120) culture, and placed in a 37°C, 5% CO_2_ cell incubator. DEX (Cat. No. 1179333) was purchased from Sigma. Prazosin (HY‐B0193, α2 AR inhibitor) from MCE.

### 2.2 Plasmid build

The CRISPR‐Cas9 gene‐editing system was operated according to the methods of Li^12^ and Jafari.^13^ Make appropriate adjustments according to the laboratory conditions. This study used the lenti CRISPR V2 plasmid constructed by the Lentivirus vector integrating cas9 enzyme and gRNA binding region in Zhang Feng's laboratory. When designing gRNA, *Bsm*BI primer connectors that can be inserted into Lenti CRISPR V2 plasmid should also be added. After digestion and ligating, the plasmid was transformed by the DH5α receptor state and extracted. The U6 promoter of the Lenti CRISPR V2 vector was used for Sanger single‐end sequencing verification. gRNA1 Forward: 5′‐CAC CGT TCC GCC AGG AGC AGC CGT‐3′, gRNA1 Reverse: 3‘‐CAA GGC GGT CCT CGT CGG CAC AAA‐5′; gRNA2 Forward: 5′‐CAC CGC AGG AGC AGC CGT TGG CCG A‐3′; gRNA2 Reverse: 3′‐CGT CCT CGT CGG CAA CCG GCT CAA A‐3′; gRNA3 Forward: 5′‐CAC CGT TGG CCG AGG GCA GCT TTG C‐3′; gRNA3 Reverse: 3′‐CAA CCG GCT CCC GTC GAA ACG CAA A‐3′.

### 2.3 Lentiviral transduction

Twenty‐four before transfection, cells were inoculated into six‐well plates and subcultured in a DMEM medium containing 10% fetal bovine serum. Transfection was performed when the cell density reached 70%–80%. The transacted plasmid and 5 μL PEI‐Max solution (2 μg/μL) were diluted to 150 μL using OPTI‐MEM reduced serum medium, respectively. Gently blow and mix, let stand at room temperature for 5 min and mix, blow and mix again, let stand at room temperature for 15 min. The mixture was slowly added to the 6‐well plate, shaken well, cultured overnight at 37°C for 24 h, and then replaced with fresh medium. The target cells were screened by adding puromycin resistant medium until all untransfected cells died.

### 2.4 T7E1 mismatches enzyme detected gRNA activity

Genomic DNA of the sorted cells was extracted, and amplification primers containing targeted sites that were not located in the center of the amplified fragment were designed. The length of the PCR product was 527 bp. PCR products were purified with gel recovery kits. 0.5 μL T7E1 enzyme was added to the PCR product after denaturation and annealing for 30 min in a 37°C water bath. Electrophoresis was performed at 120 V constant pressure for 30 min in a freshly configured 1.8% agarose gel. After electrophoresis, the gel imager was used to take pictures. ImageJ software calculates the gray level of each strip. The product size was 527 bp, one segment was 301 bp, and one segment was 198 bp. Through verification, gRNA with the highest enzyme activity was subsequently used, and THP‐1 cells with ADRA2 gene knockout were used as the basis of cell research, denoted as THP‐1^KO^. Forward primer: 5′‐AAT CTC TCT TTA CCC ATC GGC TCT C‐3′; Reverse primer: 5′‐GAA CAC GGC GAT GAT GAC GAG‐3′.

### 2.5 MTT measured cell viability

Logarithmic THP‐1 and THP‐1^KO^ cells were collected, and 100 μL single cell suspension at a concentration of 4 × 10^3^ cells/mL was added to a 96‐well plate. After standard culture for 24 h, different doses of LPS (10 ng/mL, 100 ng/mL, and 1000 ng/mL) or DEX (0.01 µM, 0.1 µM, 1 µM) were added for treatment, and 100 μL DMEM medium was used as a negative control. Each concentration of LPS or DEX had three repores. After 48 h, MTT (0793, Amersco, USA) was used to detect cell proliferation. The detection wavelength was 562 nm, and the reference wavelength was 630 nm.

### 2.6 Establishment and treatment of inflammation model

Human THP‐1^KO^ and THP‐1 cells seeded into 96‐well flat‐bottom culture plates grew as monolayers. The next day, inflammatory cell models were induced using LPS at a concentration of 1.0 µg/mL at 37°C for 24 h under 5% CO_2_.^14^, ^15^ DEX was added at the concentration of 0.1 µM, with 5 replicate wells in each group. Namely, set up a blank control group (control); DEX group (0.1 µg/mL DEX); LPS group (1.0 µg/mL LPS); DEX + LPS group (DEX + LPS). Subsequently, all of them were incubated at 37°C and 5% CO_2_ for 24 h.

### 2.7 ELISA detection of inflammatory factor expression

THP‐1 and THP‐1^KO^ cells were cultured and grouped in 6 cm culture dishes. The cell supernatant was collected after the cells reached 70% confluence (approximately 24 h). DEX and LPS were then added for intervention, and the cells were further cultured for 24 h. The supernatant from each group was aspirated and centrifuged for 10 min at 6000 rpm and 4°C. The levels of inflammatory cytokines in the cells treated with DEX and LPS were detected using the ELISA method. IL‐6 (SEKH‐0013), IL‐8 (SEKH‐0016), IL‐10 (SEKH‐0018), and MCP‐1 (SEKH‐0236) ELISA test kits were purchased from Solarbio.

### 2.8 Real‐time PCR detection

Sequences of β2 AR, IL‐6, IL‐8, IL‐10 and MCP‐1 were searched on the NCBI website and corresponding primers were designed. After the primer sequences are designed, they will be sent to synthesize primers for Suzhou Genewiz Biotechnology Co., Ltd. Collect the cultured cells of each group, extract the total RNA of the cells with Trizol, and reverse‐transcribed to cDNA using MMLV reverse transcriptase. Using each group of cDNA as a template, SYBR Green as a fluorescent dye, and GAPDH as the internal control, PCR reactions were performed on the FAST 7500 fluorescence quantitative PCR instrument. Three replicate wells were set for each sample. The relative expression of genes in each group was calculated by the $△△C_{T}$ method. The calculation formula is as follows: $2^{-△△C_{t}}$ $△△C_{T}$ = (*C*_T_ experimental group‐*C*_T_ experimental group internal reference)‐(*C*_T_ control group‐*C*_T_ control internal reference). ADRB2, forward: 5′‐TAC CAG AGC CTG CTG ACC AAG A‐3′, reverse: 5′‐AGT CAC AGC AGG TCT CAT TGG C‐3′; ADRA2A, forward: 5′‐CTT CTG GTT CGG CTA CTG CAA C‐3′, reverse: 5′‐GGA AAC CTC ACA CGA TCC GCT T‐3′; ADRA2, forward: 5′‐GCT TGG CAG AGA GAT AGC CG‐3′, reverse: 5′‐AAA ACC CTG ACC TCA CAG CC‐3; GAPDH: forward: 5′‐GTC TCC TCT GAC TTC AAC AGC G‐3′; reverse: 5′‐ ACC ACC CTG TTG CTG TAG CCA A‐3′.

### 2.9 Western blot

Take the cultured cells, discard the culture medium, place the culture dish on ice, rinse the cells twice with cold PBS, aspirate the remaining PBS, lyse the cells on ice with RIPA lysate, and blow with a 1 mL syringe to break the cell DNA. After incubating at 95°C for 10 min, take 3 μL and quantify with BCA. After boiling the protein and the loading buffer, store them at −20°C until use. Prepare SDS‐PAGE electrophoresis solution and add boiled samples for electrophoresis. After electrophoresis, transferred the protein to the PVDF membrane with a constant current of 300 mA for 1.5 h, and blocked with 5% skimmed milk. Primary antibodies (Table 1) are added and incubated overnight at 4°C. The HRP‐labeled goat anti‐rabbit IgG antibody is added and incubated for 1 h at 25°C. After that, wash the membrane 3 times with TBST, remove the membrane, add ECL luminescent solution, and press in the dark room development.

**Table 1:** Antibody information.

Name	Company	Catalog number	Dilution
PKA	Huabio Company	ER64617	1:500
p‐PKA	Cell SignalingTechnology	#38938	1:500
p‐CREB	Abcam	ab32096	1:500
CREB	Abcam	ab178322	1:500
p‐NF‐κB	Huabio Company	ET1604‐27	1:500
NF‐κB	Huabio Company	ET1603‐12	1:500
GAPDH	Huabio Company	ET1601‐4	1:1000
HRP‐labeled goat anti‐rabbit IgG secondary antibody	Huabio Company	HA1001	1:2000
β2 AR	Abcam	ab176490	1:500
ADRA2	Huabio Company	ER62703	1:500

### 2.10 siRNA interferes with β2 AR and α2 AR expression

The BLAST provided by NCBI GenBank was used for siRNA design (http://blast.ncbi.nlm.nih.gov/Blast.cgi) and entrusted to Genwiz Biotechnology Co., Ltd.(Suzhou, China) for synthesis. Adopts cells in the logarithmic growth phase, which is 1/3 of the number of conventionally cultured cells for transfection experiment. Take 0.67 μg (50 pmol) siRNA, add a certain amount of serum‐free diluent, and mix well to make RNA diluent, the final volume is 25 μL. The above dilution and RNA dilution were mixed thoroughly (can be shaken with a shaker or pipetted more than 10 times with a sampler), and allowed to stand at 25°C for 15 min. Drop 50 μL of the transfection complex onto cells added with 0.45 ml of complete medium (containing 10% serum and antibiotics), move the petri dish back and forth, and mix well. 6 h after transfection, the complete medium was changed. β2 AR‐siRNA1: Forward 5′‐AUU UUC AUA AGA AUA UGG GCG‐3′, Reverse 5′‐CCC AUA UUC UUA UGA AAA UGU‐3′; β2 AR‐siRNA2: Forward 5′‐ACA UUU UCA UAA GAA UAU GGG‐3′, Reverse 5′‐CAU AUU CUU AUG AAA AUG UGG‐3′; β2 AR‐siRNA3: Forward 5′‐AAA UAA CAA AUA AUC ACU CAA‐3′, Reverse 5′‐GAG UGA UUA UUU GUU AUU UGU‐3′. α2 AR‐siRNA1: Forward 5′‐GGC CCG CTC TTC AAG TTC TTC TTC T‐3′; Reverse 5′‐GGC CGC TTT ACG ATT TCC TTC CTC T‐3′; α2 AR‐siRNA2: Forward 5′‐CCG CTC TTC AAG TTC TTC TTC TGG A‐3′; Reverse 5′‐CCG CTT CTG AAT TCT CTT CTT CGG A′; α2 AR‐siRNA3: Forward 5′‐CAA CCA GGA TTT CCG GCG ATC CTT T‐3′; Reverse 5′‐ CAA GAA GTT CTG CCG AGC TCC CTT T‐3′.

### 2.11 Immunofluorescence

For monolayer growth cells, when subcultured, inoculate the cells in a Petri dish containing treated coverslips in advance, take out the coverslips after the cells are nearly grown into a monolayer, wash with PBS twice and use 4% paraformaldehyde‐fixed. Permeabilize cells at room temperature and then block antigens, incubating with β2AR rabbit anti‐human primary antibody (1:50) overnight at 4°C. Incubate with a secondary antibody with fluorescent‐labelled and HRP‐labeled goat anti‐rabbit IgG. Then, DAPI was used for nuclear staining for cell localization. After rinsing with ddH_2_O, a mounting tablet containing an anti‐fluorescence quencher was added dropwise on the glass slide for mounting. Observe with a fluorescence microscope (DM500, Leica).

### 2.12 Molecular docking

Downloaded the 3D structure of DEX (Compound CID: 5311068) in the PubChem database (https://pubchem.ncbi.nlm.nih.gov/), and used the MMFF94 force field for optimization. The 3D structure of adrenergic receptor beta2 (Beta‐2 adrenergic receptor, ADRB2, β2 AR) was downloaded from Protein Data Bank (www.rcsb.org, PDB ID: 6NI3). Used AutodockTools1.5.6 to search and define the compound DEX for adding hydrogen atoms, removing water, adding hydrogen atoms and adding charges to the ADRB2 structure, and saving them as PDBQT format. Used Autodockvina 1.1.2 to molecular docking. To increase the accuracy of the calculation, here, set the parameter exhaustiveness to 20. All other parameters use default values. In the end, the conformation with the highest score is selected to analyze the results with Pymol.

### 2.13 Statistical methods

All statistical data are analyzed with SPSS 22.0, and measurement data are expressed as mean ± standard deviation (x ± s). The Shapiro‐Wilk test was used to assess the normal distribution of the samples. For data that followed a normal distribution, differences were analyzed using one‐way ANOVA followed by Tukey's post hoc test. For non‐normally distributed data, the Kruskal−Wallis test was used for analysis. The LSD‐t test was used to compare the mean between the two groups. *p* < .05 was considered statistically significant.

## 3 RESULTS

### 3.1 Effect of DEX and LPS on THP‐1 activity and expression of cytokines

**Figure 1:**
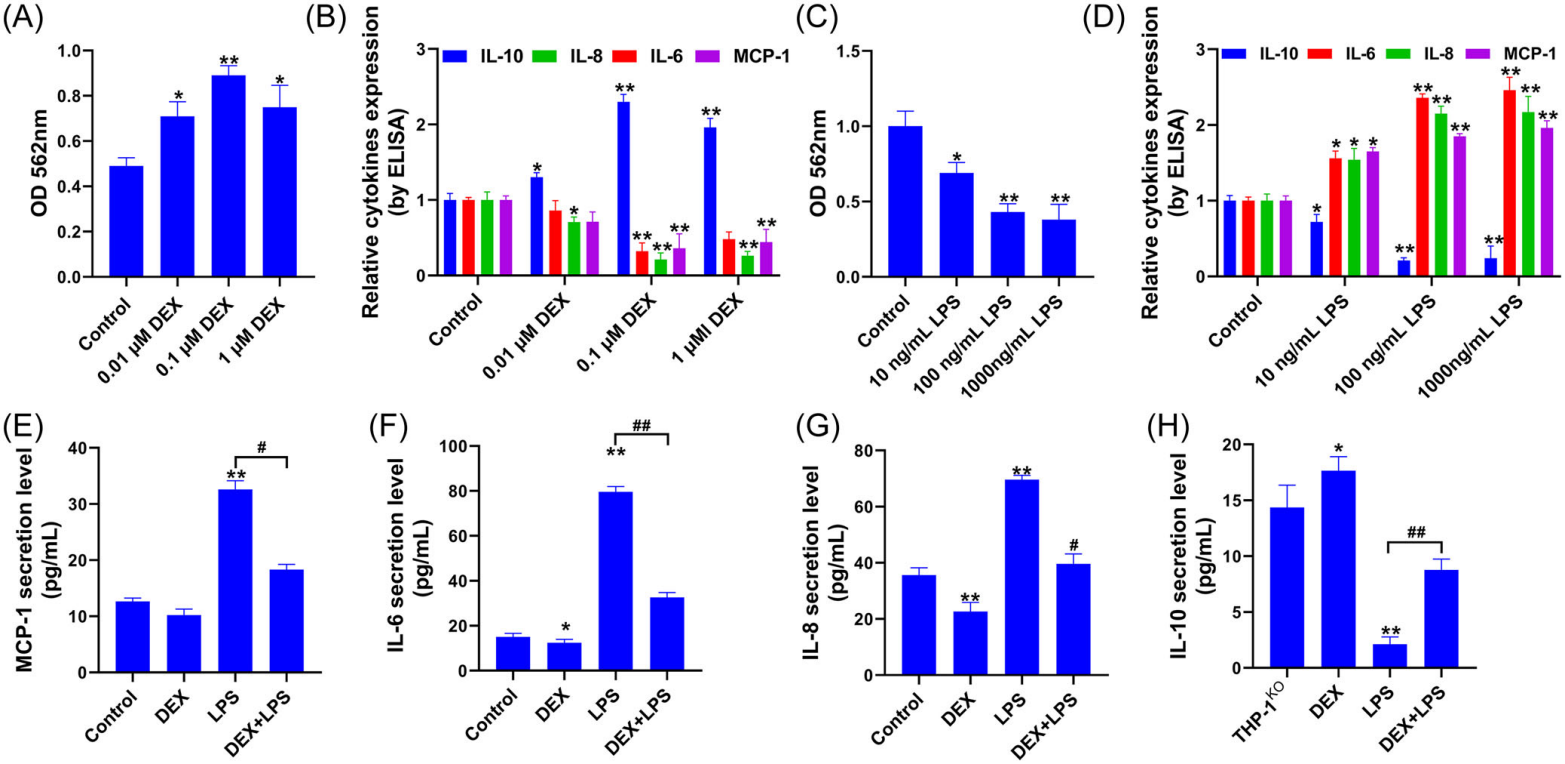
Effect of DEX on THP‐1 inflammatory cytokines secretion level. (A) MTT detected the effects of 0, 0.01, 0.1,1 μM DEX on cell proliferation. (B) ELISA was used to detect the protein secretion levels of IL‐10, IL‐6, IL‐8 and MCP‐1 after 0, 0.01, 0.1 and 1 μM DEX treatment. (C) MTT assay was used to detect the effects of 0, 10, 100 and 1000 ng/mL LPS on cell proliferation. (D) ELISA was used to detect the secretion levels of IL‐10, IL‐6, IL‐8 and MCP‐1 after LPS treatment at 0, 10, 100 and 1000 ng/mL. (E−H) Protein secretion levels of MCP‐1, IL‐6, IL‐8 and IL‐10 in cell supernatant were detected by ELISA. **p < .01, *p < .05, compared with the control group. ^##^ *p* < .01, ^#^ *p* < .05, the two groups of wired were compared.

Figure 1A shows the effect of MTT on THP‐1 cell viability under the conditions of 0, 0.01, 0.1, and 1 μM DEX. The results showed that the cell proliferation activity was the highest under 0.1 μM DEX, and the difference was statistically significant compared with the control group (*p* < .01). ELISA assay of inflammatory cytokine releases (Figure 1B) showed that IL‐10 anti‐inflammatory cytokine secretion significantly increased after 0.1, 1 μM DEX treatment compared with the control group (*p* < .05). IL‐8, IL‐6, and MCP‐1 cytokine secretion were significantly inhibited compared with the control group (*p* < .05), and there was no significant difference between the 0.1 μM DEX group and 1 μM DEX group on the regulation level of cytokines (*p* > .05). Figure 1C shows the proliferation activity of THP‐1 cells detected by MTT in the presence of LPS at 0, 10, 100, and 1000 ng/mL. The results showed that compared with 10 ng/mL, 100 ng/mL and 1000 ng/mL LPS upregulated the cytokine secretion levels of IL‐6, IL‐8, and MCP‐1, and decreased the cytokine secretion levels of IL‐10, with significant difference between the two groups (*p* < .01). There was no significant difference between 100 ng/mL and 1000 ng/mL LPS groups (*p* > .05). Therefore, in subsequent experiments, 0.1 μM DEX and 100 ng/mL LPS were used as the working concentration. Figure 1E−H shows the ELISA results of inflammatory index MCP‐1, IL‐6, IL‐8, and anti‐inflammatory index IL‐10, respectively. The results showed that compared with the control group, the protein expression levels of MCP‐1, IL‐6, and IL‐8 in THP‐1 cells were significantly increased after LPS treatment. The expression level of IL‐10 protein in THP‐1 cells was significantly decreased (*p* < .01). After adding 0.1 μM DEX, the secretion levels of MCP‐1, IL‐6, and IL‐8 decreased significantly, and IL‐10 increased significantly, with statistically significant differences compared with the LPS group (*p* < .05).

### 3.2 Inhibitory effects of α2 AR on THP‐1 DEX sensitivity in THP‐1 cells

**Figure 2:**
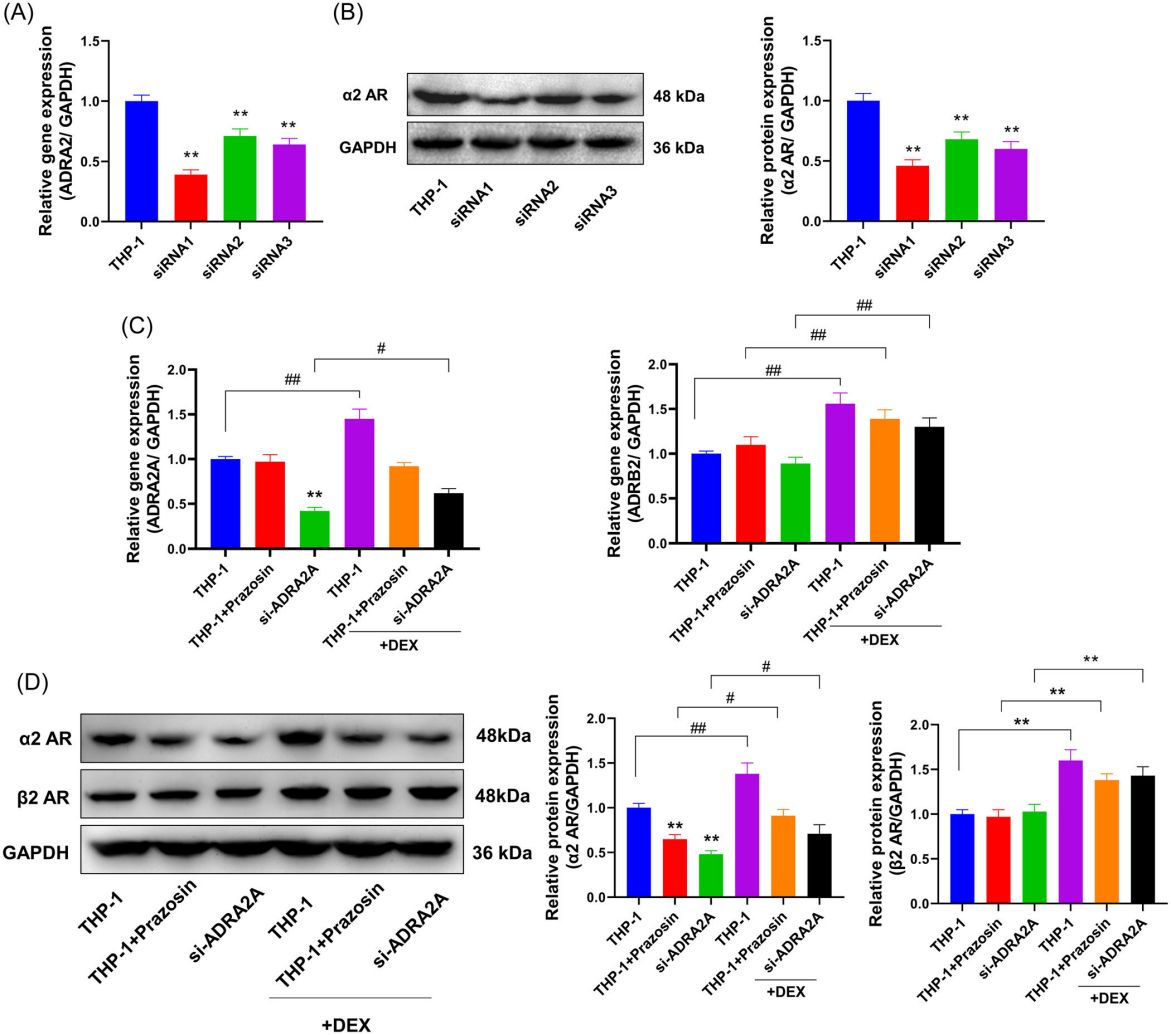
Inhibitory effects of α2 AR on DEX sensitivity in THP‐1 cells. (A) The expression level of ADRA2A gene was detected by qPCR. (B) The expression level of α2 AR protein was detected by western blot. (C) The expression level of ADRA2A and ADRB2 gene was detected by qPCR. (D) The expression level of α2 AR and β2 AR protein was detected by western blot.

To verify the impact of inhibiting α2 AR expression on DEX sensitivity, we first downregulated the ADRA2A gene in THP‐1 cells. The results showed that siRNA exhibited the highest efficiency in silencing the ADRA2A gene and α2 AR, making it suitable for subsequent studies (Figure 2A,B). Analysis of gene expression in the different cell groups (Figure 2C) indicated that the α2 AR inhibitor Prazosin did not significantly suppress ADRA2A gene expression (*p* > .05), but had a significant effect on α2 AR protein levels (*p* < .01). Upon DEX treatment, the si‐ADRA2A group showed an increase in ADRA2A and ADRB2 gene expression (*p* < .05) (Figure 2D). Protein analysis results revealed that both the Prazosin and si‐ADRA2A groups exhibited a significant decrease in α2 AR protein expression (*p* < .01). At the same time, DEX treatment significantly increased both α2 AR and β2 AR expression (*p* < .05). Therefore, knocking down the ADRA2A gene is necessary to eliminate the influence of α2 AR.

### 3.3 CRISPR was used to knock out *ADRA2A* gene in THP‐1 cells

**Figure 3:**
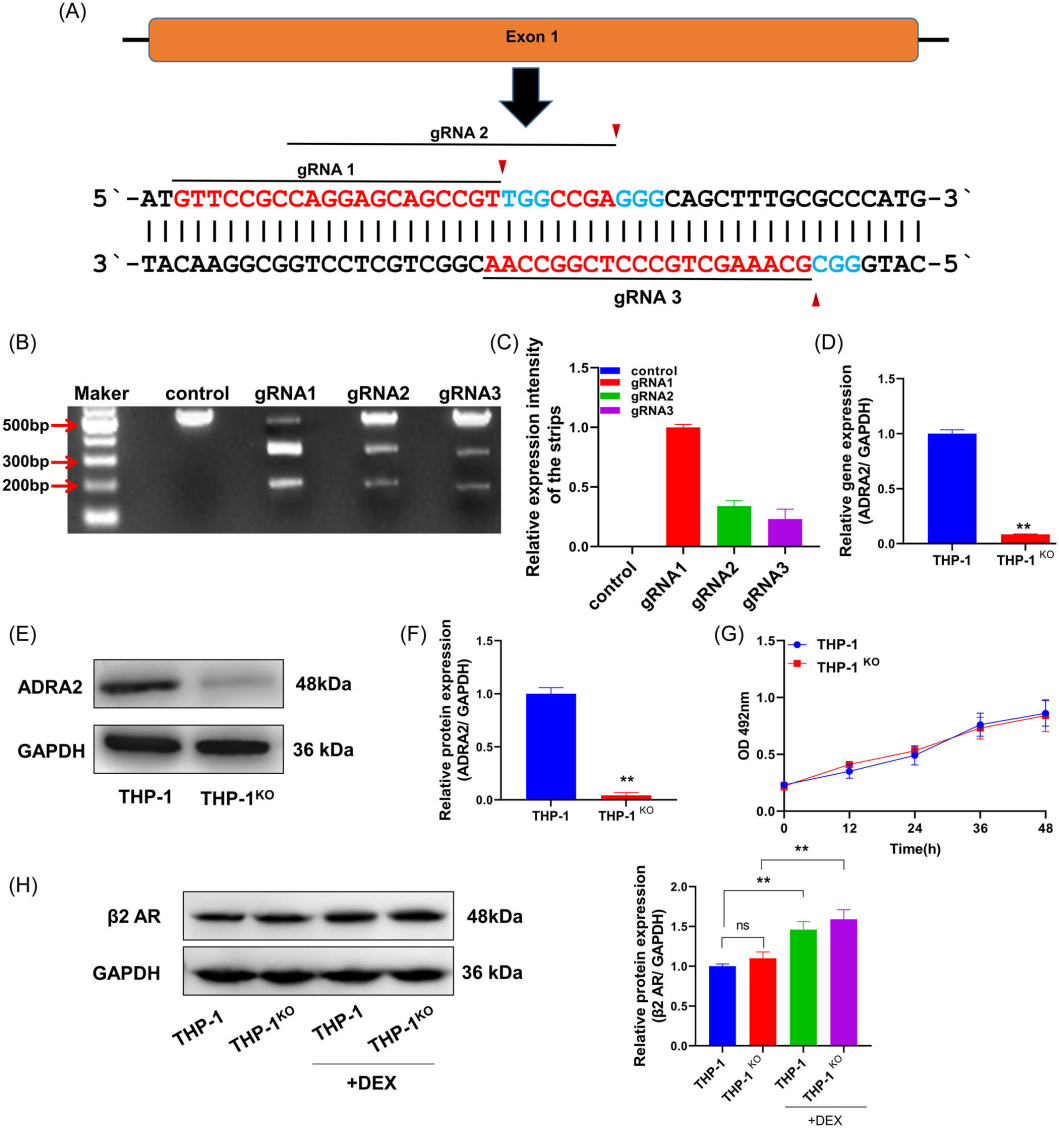
Construction of ADRA2A knockout THP‐1 cells using CRISPR technology. (A) gRNA design site of ADRA2A gene. (B) PCR amplification products were digested by T7E1 mismatch enzyme and agarose gel electrophoresis was performed. (C) Results of statistical quantization of optical density of electrophoretic strip. (D) The expression level of ADRA2A gene was detected by qPCR. (E) The expression level of α2 AR protein was detected by western blot. (F) Western blot analysis results were statistically quantified. (G) MTT was used to detect cell proliferation activity. (H) β2 AR protein expression after the ADRA2A knockout, and treated with DEX. **p < .01, compared with the control group or THP‐1 group.

The CRISPR gRNA locus was designed according to the CDS region of the ADRA2A gene. As shown in Figure 3A, 3 gRNAs were designed, and blue is the sequence of TARGETED sites for gRNA NGG. Detection was performed after lentivirus infection with lenti CRISPR V2 shRNA 1‐3 plasmid. Figure 3B shows the enzyme digestion of the PCR amplification band of the ADRA2A gene by the T7E1 mismatch enzyme. The results show that gRNA1 has a higher cleavage activity on the ADRA2A gene, and the cleavage band after T7E1 mismatch enzyme digestion is the brightest at about 300 and 200 bp (Figure 3C for the quantitative results of optical density value). Therefore, Lenti CRISPR V2‐gRNA1 was used as an ADRA2A knockout infection plasmid to construct ADRA2A knockout THP‐1 cells, denoted as THP‐1^KO^. qPCR results showed (Figure 3D) that compared with wild‐type THP‐1 cells, the expression level of the ADRA2A gene in THP‐1^KO^ cells was significantly reduced, and the difference was statistically significant (*p* < .05). Western blot analysis results showed (Figure 3E,F) that the expression level of α2 AR protein in the THP‐1^KO^ group was significantly decreased compared with THP‐1 cells, and the difference was statistically significant (*p* < .01). MTT results showed that ADRA2A knockdown had no significant effect on THP‐1 cell proliferation (Figure 3G, *p* > .05). After ADRA2A knockout, although the expression of β2 AR in the THP‐1^KO^ group was elevated compared to THP‐1, there was no statistical difference (Figure 3H, *p* > .05), and after the addition of DEX, the expression of β2 AR was significantly increased (*p* < .01).

### 3.4 Effect of DEX on the secretion of THP‐1^KO^ cytokines

**Figure 4:**
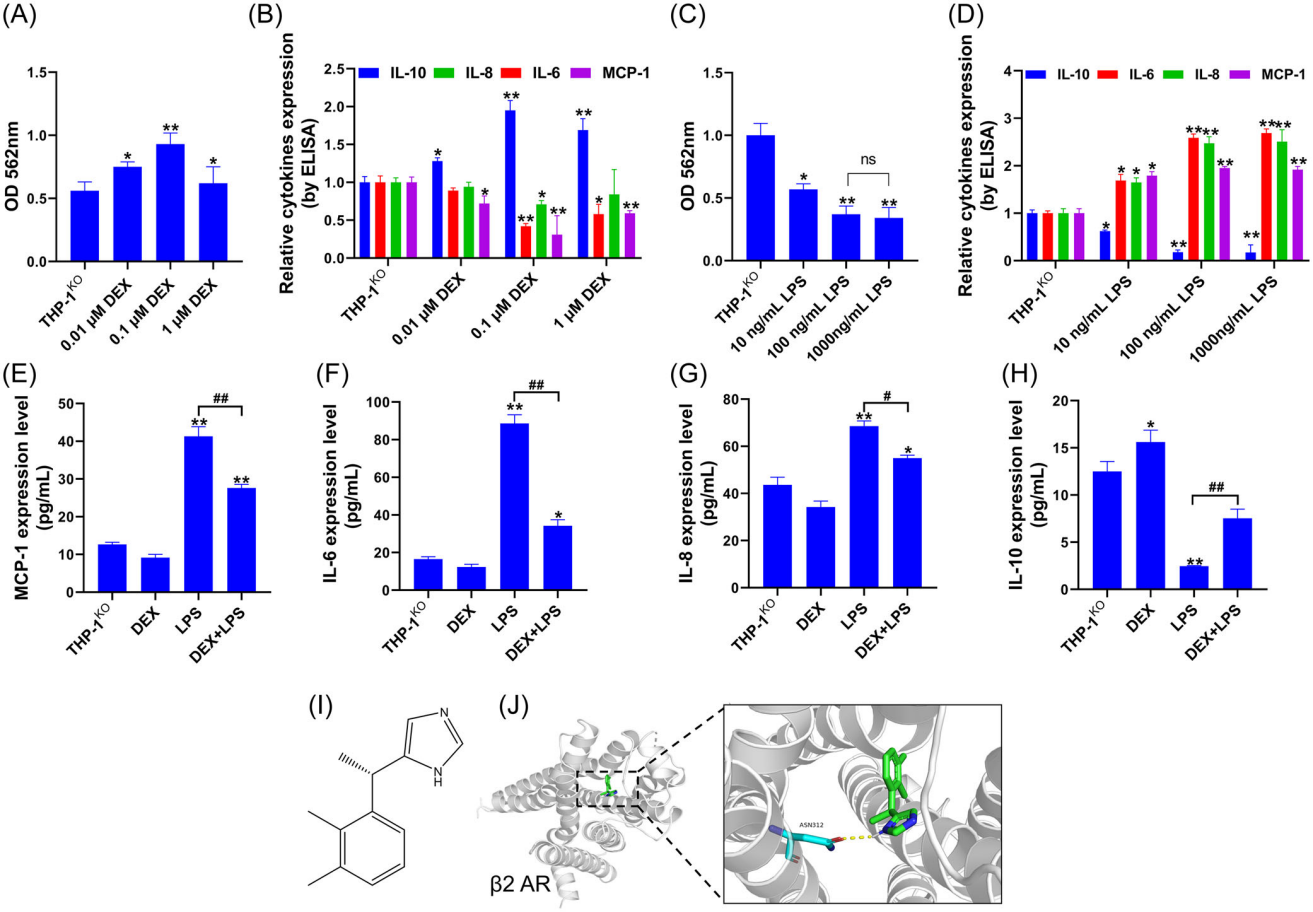
Effect of DEX on secretion of THP‐1 ^KO^ inflammatory factors. (A) MTT detected the effects of 0, 0.01, 0.1, 1 μM DEX on cell proliferation. (B) ELISA was used to detect the protein secretion levels of IL‐10, IL‐6, IL‐8, and MCP‐1 after 0, 0.01, 0.1, and 1 μM DEX treatment. (C) MTT assay was used to detect the effects of 0, 10, 100 and 1000 ng/mL LPS on cell proliferation. (D) ELISA was used to detect the secretion levels of IL‐10, IL‐6, IL‐8, and MCP‐1 after LPS treatment at 0, 10, 100, and 1000 ng/mL. (E‐H) Protein secretion levels of MCP‐1, IL‐6, IL‐8, and IL‐10 in cell supernatant were detected by ELISA. (I) DEX 2D structural formula. (J) DEX and β2 AR protein were simulated by Pymol software. **p < .01, *p < .05, compared with the control group. ^##^ *p* < .01, ^#^ *p* < .05, the two groups of wired were compared.

First, we analyzed the cytotoxicity of DEX in THP‐1^KO^ cells, and the results showed that 0.1 μM DEX treatment significantly increased THP‐1^KO^ proliferation activity (*p* < .01, Figure 4A). Figure 4B shows the secretion of IL‐10, IL‐6, IL‐8, and MCP‐1 detected by ELISA. The results showed that IL‐6, IL‐8, and MCP‐1 proteins were significantly decreased under 0.1 μM DEX treatment, and IL‐10 protein secretion was significantly increased (*p* < .01). Figure 4C shows the effect of different doses of LPS on the THP‐1^KO^ cell proliferation activity of MTT. Compared with the THP‐1^KO^ group, the results showed that both 100 and 1000 ng/mL LPS significantly decreased the proliferation activity of THP‐1^KO^ cells (*p* < .01). There was no significant difference in the proliferation activity of THP‐1^KO^ cells between 100 and 1000 ng/mL LPS (*p* > .05). The effects of different doses of LPS on THP‐1^KO^ cell inflammatory indexes were detected by ELISA (Figure 4D). The results showed that the IL‐6, IL‐8, and MCP‐1 secretion levels in THP‐1^KO^ cells were significantly upregulated after 100 and 1000 ng/mL LPS. LPS also inhibited IL‐10 secretion, and the difference between the two groups was statistically significant compared with the THP‐1^KO^ group (*p* < .05). In summary, 0.1 μM DEX and 100 ng/mL LPS will be used as the primary research done in the subsequent experiments. Figure 4E−H showed that the LPS + DEX group could significantly inhibit the LPS‐induced increase of IL‐6, IL‐8, and MCP‐1 secretion after DEX administration. At the same time, the protein secretion level of IL‐10 was increased, and the difference was statistically significant compared with the LPS group (*p* < .05). Figure 4I is the schematic diagram of the DEX structure. DEX was docked with β2 AR active pocket with a binding energy of −7.4 kcal/mol. As can be seen from the Figure 4J, the composite DEX presents a compact binding mode in the active pocket. Detailed analysis shows that DEX forms a hydrogen bond with amino acid ASN312, enabling DEX and β2 AR to form a stable complex.

### 3.5 DEX regulates THP‐1^KO^ cytokine expression through β2 AR/PKA pathway

**Figure 5:**
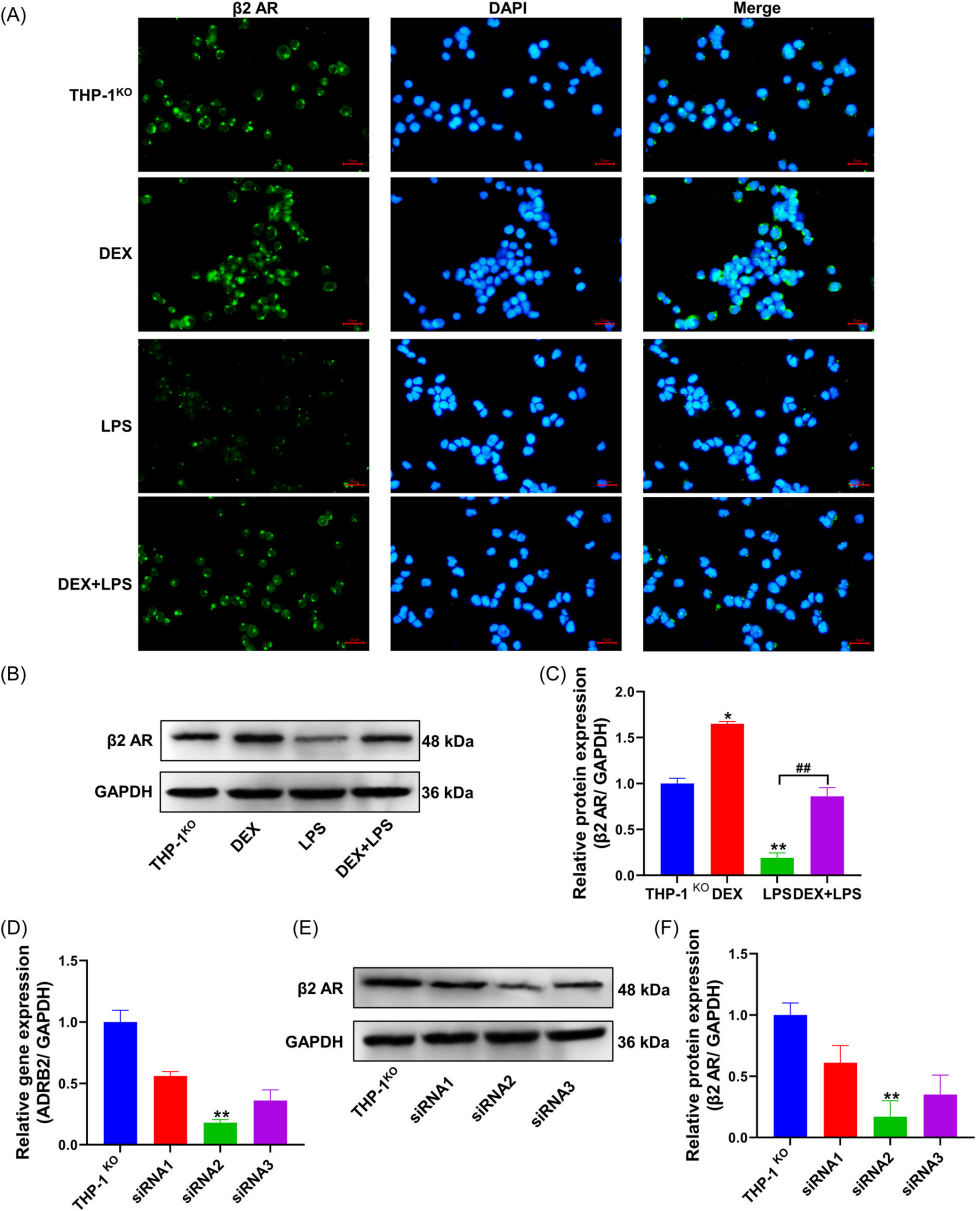
DEX promotes the expression of β2 AR. (A) The distribution and expression level of β2 AR protein were detected by immunofluorescence. (B) The expression level of β2 AR protein was detected by western blot. (C) Statistical quantification results of optical density values of western blot bands. (D) qPCR was used to detect the expression level of ADRB2 gene in siRNA1‐3 transfected cells. (E) The expression level of β2 AR protein in siRNA1‐3 transfected cells was detected by western blot. (F) Results the optical density of the western blot bands in E was statistically quantified. **p < .01, *p < .05, compared with THP‐1^KO^ group. ^##^ *p* < .01, ^#^ *p* < .05, the two groups of wired were compared.

The distribution and expression of β2 AR protein in THP‐1^KO^ cells were detected by immunofluorescence (Figure 5A), and the results showed that β2 AR protein was mainly distributed in the cell membrane and cytoplasm in THP‐1^KO^ cells. After exposure at 500ms, the fluorescence intensity of β2 AR protein in THP‐1^KO^ cells increased after DEX treatment. It decreased significantly after LPS treatment, while the positive expression intensity of β2 AR protein increased in the DEX + LPS group. The distribution of β2 AR protein did not change after LPS or DEX treatment. Combined with Western blot analysis results (Figure 5B,C), LPS treatment significantly inhibited β2 AR protein expression compared with the THP‐1^KO^ group (*p* < .01). To explore whether DEX plays an anti‐inflammatory role through upregulation of β2 AR protein, we combined siRNA for further study. Figure 5D shows the expression level of the ADRB2 gene in THP‐1^KO^ cells transfected with siRNA 1‐3 detected by qPCR. The results showed that the expression level of the ADRB2 gene in THP‐1^KO^ cells was significantly inhibited after transfection with siRNA2, the difference was statistically significant compared to control THP‐1^KO^ cells (*p* < .01), and the inhibition rate was 82%. After siRNA 1−3 was transfected with THP‐1^KO^ cells, the expression level of β2 AR protein was detected by Western blot (Figure 5E,F).

**Figure 6:**
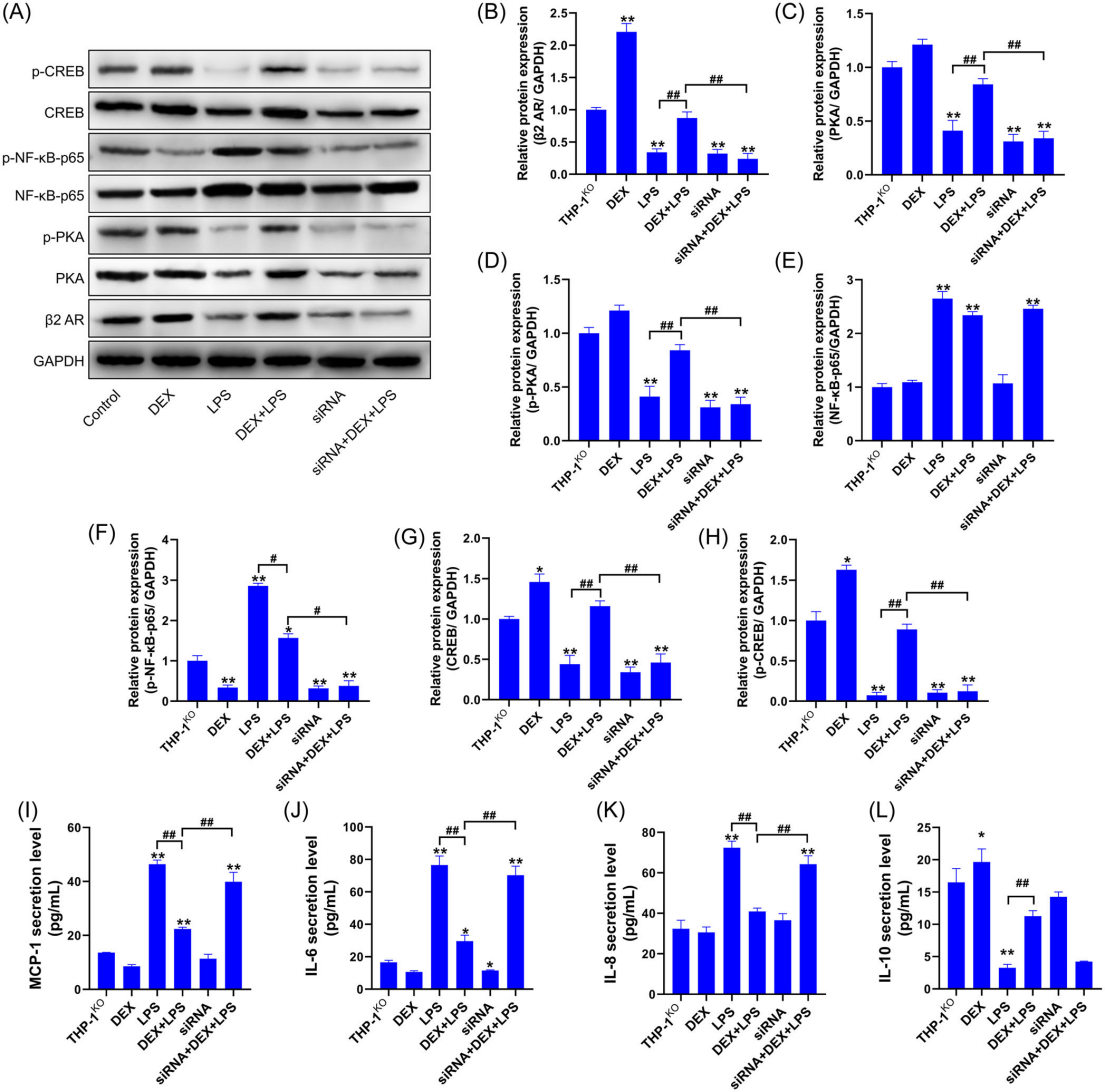
DEX regulates inflammation by regulating β2 AR/PKA pathway. (A) The protein expression levels of β2 AR, PKA, NF‐κB, and CREB and the phosphorylation levels of PKA, NF‐κB, and CREB were detected by Western blot analysis. (B−H) Western blot analysis results showed that the optical density of the bands was statistically quantified. (I‐L) Protein secretion levels of McP‐1, IL‐6, IL‐8 and IL‐10 in cell supernatants were detected by ELISA. **p < .01, *p < .05, compared with THP‐1^KO^ group. **p < .01, *p < .05, compared with THP‐1^KO^ group.

The results showed that the expression level of β2 AR protein in THP‐1^KO^ cells was significantly inhibited after transfection with siRNA2 (*p* < .01). In summary, we used siRNA2 as the follow‐up experimental siRNA of ADRB2. The protein expression levels of β2 AR, PKA, NF‐κB, and CREB and the phosphorylation levels of PKA, NF‐κB, and CREB were detected by Western blot analysis (Figure 6A−H). The results showed that compared with the THP‐1^KO^ group, β2 AR/PKA/CREB pathway protein expression level and phosphorylation level were significantly inhibited after LPS supplementation (*p* < .05), NF‐κB and its phosphorylation level were significantly increased (*p* < .05). Compared with the LPS group, β2 AR/PKA/CREB pathway protein expression and phosphorylation level were increased in the DEX + LPS group (*p* < .05), and NF‐κB phosphorylation was significantly inhibited (*p* < .05). The enhanced effect of DEX + LPS on β2 AR/PKA/CREB pathway protein expression and phosphorylation and the inhibitory effect of NF‐κB phosphorylation were significantly reversed after siRNA transfection. The difference between the two groups was statistically significant (*p* < .05). At the same time, ELISA was performed on inflammatory indicators, and the results showed (Figure 6I−L) that after siRNA transfection, the inhibitory effect of DEX on proinflammatory factors MCP‐1, IL‐6 and IL‐8, as well as the secretion enhancement effect of anti‐inflammatory factor IL‐10 protein was reversed.

## 4 DISCUSSION

DEX is a highly selective α2 AR agonist that exhibits sedative properties, hemodynamic stability, analgesia, reduced anesthetic requirements, anti‐anxiety effects, sympathetic inhibition, and mild respiratory depression. Moreover, DEX has been reported to possess anti‐inflammatory properties and can attenuate inflammatory damage associated with various diseases.^16^ Of particular interest is its potential to reduce LPS‐mediated inflammation, as LPS has been demonstrated to induce the release of inflammatory cytokines from monocytes, including THP‐1 cells.^17^, ^18^

A meaningful relationship exists between the balance of inflammatory factors and various cytokines in the body and immune response. Different cytokines can play different immune effects, such as anti‐inflammatory cytokines IL‐2 and IL‐10, which mainly inhibit the release of inflammatory mediators and prevent excessive inflammatory reactions in the body.^19^ IL‐6, IL‐8, MCP‐1, and other proinflammatory factors mainly mediate inflammatory responses, leading to tissue damage.^9^, ^20^, ^21^ Cytokines are involved in initiating and regulating immune response and inflammatory response.^20^ This study employed a LPS‐induced inflammatory injury model using human THP‐1 cells. The findings demonstrated that DEX intervention decreased the expression of MCP‐1, IL‐6, and IL‐8. Additionally, the expression of IL‐10 increased following DEX treatment in the context of LPS‐induced inflammatory injury. These results indicate that DEX can potentially reduce the release of proinflammatory factors, elevate the levels of anti‐inflammatory factors, and attenuate the LPS‐induced inflammatory response.

Because DEX is an α2‐AR agonist, we constructed ADRA2A gene knockout THP‐1 cells using CRISPR technology for study. The CRISPR‐Cas9 gene‐editing system is beneficial in cell biology, providing a way to knock out specific genes in human cell lines.^22^ In addition, effects evoked by CRISPR/Cas9 mediated gene deletion of A2AR are superior to shRNA‐mediated knockdown or pharmacological blockade of A2AR.^23^ In this report, we generated ADRA2A knockout THP‐1 cell lines using the CRISPR‐Cas9 gene‐editing system. Our study did not use transient transfection of small interfering RNA‐mediated knockdown, which avoids potential side effects caused by transfection agents or ectopic RNA expression. This study showed that the knockout of ADRA2A did not induce significant changes in THP‐1 proliferation activity. At the same time, treatment with DEX after ADRA2A deletion still significantly reduced LPS‐induced THP‐1^KO^ cell inflammatory cytokines. Therefore, we inferred that DEX attenuates the inflammatory response of THP‐1 in other ways than through the α2 AR pathway. This effect may be independent of α2‐adrenergic receptors, but the mechanism of DEX regulation of cytokines remains to be elucidated.

Activation of β2 AR has been reported to down‐regulate the release of proinflammatory cytokines in human bronchial epithelial cells. Many studies have also demonstrated that β2 AR expresses inflammatory factors.^24^, ^25^ Activation of β2 AR has also been shown to inhibit NF‐κB pathway activation and down‐regulate TLR4‐mediated inflammatory response in THP‐1 cells.^25^, ^26^ Therefore, this study docked the binding site of DEX to β2 AR protein agonist and found that DEX could dock with the active pocket of β2 AR through β2AR ASN312 hydrogen bond, which provided a certain possibility for the binding of the two. Combined with immunofluorescence and western blot experiments, β2 AR expression was increased in THP‐1^KO^ cells treated with DEX. The expression of the ADRB2 gene and protein was further inhibited by siRNA. The results showed that DEX treatment did not significantly change the total protein of NF‐κB, but significantly inhibited the phosphorylation level of NF‐κB. DEX also enhanced the expression and phosphorylation of PKA/CREB protein, which was reversed after ADRB2 siRNA was administered. β2 AR has inhibited LPS‐induced NF‐κB activation by β ‐Arrestin‐2 in THP‐1 cells.^27^ In Th17 cells, β2 AR agonists increased PKA expression and activity, decreasing inflammatory response.^25^ In norepinephrine (NE)‐mediated anti‐inflammatory studies of THP‐1 cells, NE has been shown to modulate innate inflammatory responses induced by amyloid‐beta peptides by activating the PKA/CREB pathway.^28^ Lima et al.^29^ also demonstrated that cyclic AMP alleviates acute inflammation mainly by activating the PKA/CREB pathway and increasing the expression of conjunction A1 to achieve anti‐inflammatory effects. In addition, phosphorylation of CREB directly inhibits NF‐κB activation by blocking the binding of CREB‐binding protein to the NF‐κB complex, thereby limiting the proinflammatory response.^30^ Combined with the results of this study, after the addition of DEX, the PKA/CREB pathway was activated in THP‐1^KO^ cells, and the expressions of proinflammatory factors IL‐6, IL‐8, and MCP‐1 were inhibited, while the expression of anti‐inflammatory factor IL‐10 was enhanced. After inhibition of ADRB2 gene expression, the activation level of the β2 AR/PKA/CREB pathway was significantly inhibited, and the expression of inflammatory factors was increased while that of anti‐inflammatory factors was decreased.

In conclusion, DEX may activate the PKA/CREB pathway by activating β2 AR, inhibiting NF‐κB activation. This, in turn, reduces the transcription of proinflammatory factors while increasing the transcription of anti‐inflammatory factors. The findings of this study provide scientific evidence for the use of DEX in the treatment of inflammation and offer insights for further exploring its potential applications.

## AUTHOR CONTRIBUTIONS

**Baocheng Zhang**: Conceptualization, data curation, investigation, methodology, project administration, writing—review and editing. **Jie Shen**: Conceptualization, formal analysis, methodology, visualization, writing—original draft.

## CONFLICTS OF INTEREST STATEMENT

The author declare no conflict of interest.

## Data Availability

All data generated or used during this study are included in this published article, still, further details are available when request from the corresponding author.
